# Silicic Acid in Tuberculosis and Cancer

**Published:** 1914-08

**Authors:** 


**Silicic Acid in Tuberculosis and Cancer.** H. Kahle, Muench. Med. Woch., page 752, 1914, finds this substance diminished in the pancreas in tuberculosis, increased in cancer, but less is eliminated in the urine in either disease than the normal. Its administration favors the formation of connective tissue, and encapsulation and cicardization of tuberculous foci.

